# High seroprevalence, clinical predictors, and epidemiological risk factors of *Ehrlichia canis* infection in dogs on the Northern Coast of Perú: A large-scale cross-sectional study

**DOI:** 10.14202/vetworld.2025.3968-3981

**Published:** 2025-12-18

**Authors:** Raquel Patricia Ramírez-Reyes, Liany Karina Quispe-Rodríguez, Roy Macedo-Macedo, Juan R. Paredes-Valderrama

**Affiliations:** 1 Research Hotbed in Companion Animals, Faculty of Agricultural Sciences, Veterinary Medicine and Zootechnics study program, Universidad Privada Antenor Orrego (UPAO), Trujillo, Perú; 2 Animal Science Research Unit, Faculty of Agricultural Sciences, Veterinary Medicine and Zootechnics study program, Universidad Privada Antenor Orrego (UPAO), Trujillo, Perú

**Keywords:** canine ehrlichiosis, clinical signs, *Ehrlichia canis*, hematological alterations, Perú, *Rhipicephalus sanguineus*, risk factors, seroprevalence, stray dogs, tick-borne disease

## Abstract

**Background and Aim:**

Canine monocytic ehrlichiosis, caused by *Ehrlichia canis* and transmitted primarily by *Rhipicephalus sanguineus*, is a common yet diagnostically challenging tick-borne disease in tropical regions. On the northern coast of Perú, environmental conditions favor vector persistence, but local data on clinical characteristics and risk determinants remain limited. This study aimed to determine the seroprevalence of *E. canis* in domestic dogs in Trujillo (La Libertad, Perú), describe associated clinical findings, and identify epidemiological risk factors linked to infection.

**Materials and Methods:**

A cross-sectional analytical study was conducted from December 2023 to August 2024 involving 462 dogs with compatible clinical signs and/or tick infestation from 18 veterinary clinics across three districts. Serological testing was performed with the CaniV-4® rapid test, and hematological parameters were analyzed with an automated analyzer. Epidemiological data were obtained through owner questionnaires. Associations were evaluated using chi-square tests, logistic regression (Odds ratio [OR], 95% CI), and Mann–Whitney U tests for hematological differences. A p-value < 0.05 with OR and lower CI >1 defined risk factors.

**Results:**

The overall seroprevalence of *E. canis* was 51.3% (95% CI: 46.7%–55.8%). Sex and breed were not associated with infection. Dogs <1 year old (OR = 1.46), those lacking external deworming (OR = 1.99), fed homemade diets (OR = 2.26), and those frequently contacting stray dogs (OR = 4.33) were at significantly higher risk. Clinical predictors strongly associated with infection included lethargy (OR = 5.55), fever (OR = 5.52), anorexia (OR = 4.24), anemia (OR = 4.12), lymphadenopathy (OR = 3.46), and epistaxis (OR = 2.50). Seropositive dogs exhibited significantly reduced erythrocyte counts, hematocrit, hemoglobin, leukocyte counts, and platelet counts (p < 0.01). Although tick presence and park access were associated with seropositivity, their OR < 1 suggested confounding rather than true protective effects.

**Conclusion:**

The high seroprevalence and significant clinical–hematological alterations highlight widespread exposure to *E. canis* among dogs in northern coastal Perú. Identified risk factors emphasize the need for integrated tick-control, improved owner awareness, and strengthened diagnostic protocols. Future research combining molecular confirmation, socioeconomic variables, and One Health–based surveillance is recommended to refine prevention and management strategies.

## INTRODUCTION

Canine ehrlichiosis is an infectious disease caused by *Ehrlichia canis*, an intracellular bacterium that primarily affects dogs, although human infection has also been documented. Owing to its broad global distribution, it is considered one of the most commonly diagnosed vector-borne diseases in veterinary practice [1]. Prevalence varies widely between countries; for example, Colombia has reported seropositivity rates as high as 66% [2], whereas Brazil has reported 42.5%, with no clear associations with factors such as age, sex, area of residence, public exposure, or environmental conditions [3]. These variations are often linked to the distribution of the tick vector *Rhipicephalus sanguineus*, which is commonly found in dog-inhabited environments [4], particularly in regions with warm climates that favor its survival and reproductive cycle [5, 6]. Such conditions are typical of the northern coast of Perú, where temperatures fluctuate between 35°C in summer and 14°C in winter, with relative humidity ranging from 70% to 80%.

Diagnosis of the disease remains challenging due to the diversity of clinical manifestations, which vary depending on the infection stage (acute, subclinical, or chronic) and the presence of coinfections [7, 8]. Thrombocytopenia is one of the most frequent hematological findings in affected dogs; however, it is not exclusive to ehrlichiosis and can also occur in other infectious and systemic diseases. Clinical signs such as fever, anorexia, and anemia further complicate diagnosis, especially in areas where molecular diagnostic tools are limited or unavailable [9, 10]. Several studies have also identified various risk factors, including age, tick presence, and environmental conditions, that may influence the likelihood of infection [11].

Despite the recognized burden of canine ehrlichiosis worldwide, substantial knowledge gaps persist regarding its epidemiology, clinical spectrum, and risk factors in Perú, particularly along the northern coastal regions, where ecological conditions favor the continuous presence of *R. sanguineus*. Existing Peruvian studies have typically relied on small sample sizes, limited geographic coverage, or narrowly focused assessments that do not simultaneously integrate seroprevalence, clinical manifestations, hematological alterations, and environmental risk determinants. Furthermore, most available studies have not accounted for the influence of owner-related practices, socioeconomic factors, or lifestyle variables, factors that may shape disease exposure in urban and peri-urban canine populations. Diagnostic limitations also contribute to uncertainty; molecular confirmation is rarely performed in local veterinary settings, and reliance on serological tests alone may underestimate active infections or misrepresent pathogen circulation dynamics. Consequently, there remains a lack of comprehensive, large-scale, clinic-epidemiological studies capable of clarifying the true burden of infection, predictors of disease, and context-specific risk factors among domestic dogs in Trujillo and surrounding districts. These gaps hinder the formulation of effective preventive and control strategies adapted to local realities.

In response to these gaps, the present study aimed to generate robust and integrated evidence on the circulation of *E. canis* in dogs from the city of Trujillo, located on the northern coast of Perú. Specifically, the study sought to determine the seroprevalence of *E. canis* using a validated rapid diagnostic assay, to describe the clinical signs and hematological alterations associated with infection, and to identify epidemiological factors that increase disease risk within this canine population. By combining clinical examinations, hematological profiling, owner-based questionnaires, and multivariate statistical analysis, this research provides a comprehensive clinic-epidemiological characterization of the disease. The ultimate goal was to strengthen local diagnostic interpretation, guide targeted tick-control and preventive interventions, and supply baseline data that support future One Health–oriented surveillance and molecular confirmation strategies in regions where the vector remains endemic.

## MATERIALS AND METHODS

### Ethical approval

This study received formal approval from the Bioethics Committee of the Veterinary Medicine and Animal Science Program of the Faculty of Agricultural Sciences at the Universidad Privada Antenor Orrego (UPAO), Trujillo, La Libertad, under Resolution No. 500-2023-FCA-UPAO. All research procedures adhered strictly to national and international ethical principles governing the use of animals in scientific studies. Specifically, the study followed the Animal Research: Reporting of *In Vivo* Experiments 2.0 guidelines and the animal welfare recommendations established by the World Organisation for Animal Health (OIE) [12, 13], ensuring responsible conduct throughout all stages of the investigation.

Informed consent was obtained from all pet owners prior to participation, and confidentiality was maintained by assigning alphanumeric codes to each animal. No personal identifiers were collected, and access to the original database was restricted solely to the principal investigator and limited to the authorized research period. The study design ensured that no unnecessary discomfort, harm, or invasive procedures were imposed on the animals, and all clinical evaluations and blood collections were performed by qualified veterinary professionals using minimally invasive techniques. The ethical safeguards implemented throughout this research guarantee compliance with institutional, national, and international standards for animal care, data protection, and responsible veterinary research.

### Study period and location

**Figure 1:**
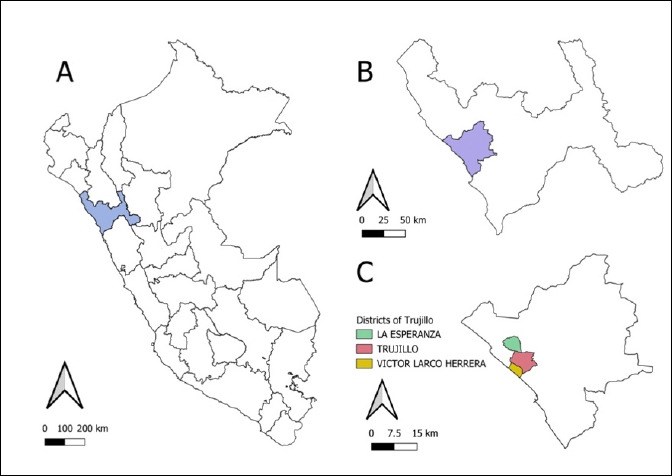
Map of the Republic of Perú and the department of La Libertad (A), province of Trujillo (B), and districts of La Esperanza, Víctor Larco Herrera, and Trujillo (C) where the study was conducted [Source: The map was generated using QGIS version 3.40.0].

This cross-sectional, observational, and analytical study was conducted between December 2023 and August 2024 in the districts of Trujillo (8°06′43′′S, 79°01′44′′W), Víctor Larco Herrera (8°08′37′′S, 79°03′22′′W), and La Esperanza (8°04′58′′S, 79°02′28′′W), located in the province of Trujillo, La Libertad, in northern Perú (Figure 1). These districts combine consolidated urban areas with expanding peri-urban areas, characterized by high population density and a large number of freely roaming semi-domesticated or stray dogs. The local ecological conditions are represented by temperatures ranging from 18°C (in autumn) to 34°C (in summer) and an average relative humidity of 76%. In peri-urban areas such as La Esperanza and some sectors of Víctor Larco Herrera, animal health management is limited, with low coverage of deworming and ectoparasite control. In contrast, the district of Trujillo features a more urban setting, with a wider availability of private veterinary care and more effective health management practices.

### Study population and sampling

A total of 462 dogs were selected from 18 veterinary clinics in the districts mentioned above, using consecutive nonprobability sampling. The sample size was determined based on a previous study that reported a prevalence of 38% [14]. Based on these results, the formula for estimating a population proportion was applied, assuming an expected prevalence of 38%, a confidence level of 95%, and an accuracy of 5%, yielding a minimum required sample size of 363 dogs. However, 462 animals were included to increase the study’s statistical power. The participating establishments were selected based on their accessibility, availability of clinical records, and willingness to collaborate with the research, ensuring adequate geographic representation and coverage across the three evaluated districts. This approach enabled the inclusion of individuals from diverse socioeconomic levels, environmental settings, and health management practices, thereby strengthening the external validity of the findings. Animals presenting clinical signs of *E. canis* infection and evidence of tick infestation were included. Dogs that had received antibiotic, antiparasitic, or anti-inflammatory treatment within 3 weeks prior to sampling were excluded, as were dogs with concomitant infectious diseases or clinical conditions that could alter hematological parameters. No restrictions regarding age, sex, or breed were applied.

### Clinical examination and diagnostic criteria

Each dog underwent a complete clinical examination and structured medical history, recording the presence or absence of the following clinical signs: lethargy, anorexia, fever, lymphadenopathy, epistaxis, lameness, uveitis, hypersalivation, and ocular discharge. In order to standardize the clinical evaluation, specific operational criteria were established for each sign: fever (rectal temperature >39.5°C), anemia (hematocrit <37% and hemoglobin <12 g/dL), lymphadenopathy (palpable enlargement of the superficial mandibular lymph nodes, prescapular lymph nodes, and inguinal lymph nodes), anemia (hematocrit <37% and hemoglobin <12 g/dL), lymphadenopathy (palpable enlargement of the superficial mandibular, prescapular, or popliteal lymph nodes compared to the anatomical standard), epistaxis (active nasal bleeding or presence of dried blood in the nostrils without other apparent cause), lethargy (marked decrease in activity, slow response, or persistent drowsiness, corroborated by the owner and clinically confirmed), anorexia (reduction or absence of food intake for more than 24 h according to the owner and clinically verified), lameness (observable gait disturbance or asymmetrical weight bearing during locomotor examination), uveitis (conjunctival redness, miosis, corneal opacity, or intraocular exudate), sialorrhea (visible hypersalivation without obvious dental cause), and ocular discharge (presence of serous or mucopurulent exudate in one or both eyes). The clinical definitions were previously agreed upon by the participating veterinarians during a training session to standardize diagnostic criteria and minimize interobserver variability. This process was conducted before the serological samples were obtained and analyzed, so that neither the veterinarians responsible for the clinical examination nor the animal owners were aware of the diagnostic test results. Therefore, the study was conducted under a simple blind design, ensuring objectivity in the clinical evaluation and interpretation of the observed signs.

### Detection of *E. canis*

Antibodies specific to *E. canis* were detected using the Anigen Rapid CaniV-4® immunochromatographic test (BioNote Inc., Suwon, South Korea). The test has reported sensitivities of 98% and specificities of 100% [14], enabling the qualitative detection of antibodies in canine serum, plasma, or whole blood. For this, 0.5 mL of blood was drawn from each dog’s cephalic vein using sterile EDTA-coated vacuum tubes (purple-top K2 vacutainers). The collected samples were stored at 4°C during transport to the laboratory and analyzed within 3 h. All serological tests were performed strictly following the manufacturer’s instructions, ensuring the presence of the control line (“C”) as an essential criterion for the test’s validity in each case. Samples that showed a clearly visible test band (“T”) within 10 to 15 min were classified as positive, even when the intensity of the line was weak, in accordance with established recommendations. Samples with indeterminate results, defined as incomplete, blurred, or difficult-to-interpret bands, underwent a second laboratory reading. The tests that remained inconclusive after this reassessment were excluded from the final analysis.

### Hematological analysis

Simultaneously, complete hematological profiles were obtained for all animals, including: erythrocyte count (10^6^/μL), hematocrit (%), hemoglobin (g/dL), mean corpuscular volume (MCV, fL), mean corpuscular hemoglobin (MCH, pg), mean corpuscular hemoglobin concentration (g/dL), leukocyte count (10³/μL), and platelet count (10^9^/L). Samples were processed using an automated hematology analyzer with leukocyte differential analysis (Mindray® BC-30 Vet, China). According to the manufacturer’s instructions, the equipment was daily calibrated prior to measurements, and accuracy was verified using internal commercial controls from the collaborating clinical laboratory. Blood samples were analyzed on the day they were collected; when immediate analysis was not possible, samples were stored at 4°C for up to 3 h before processing. Only results from samples with reading curves and counts automatically validated by the analyzer software were included in the dataset.

### Collection of epidemiological data

Epidemiological information was collected using a structured questionnaire administered to dog owners at the time of sampling. The instrument was developed by the research team and underwent content validation by three veterinarians with expertise in epidemiology and infectious diseases to ensure clarity, relevance, and consistency of each item. Before its final application, the questionnaire was pilot-tested with 20 owners, which facilitated wording adjustments and improved response efficiency. The instrument included variables related to sex, age, breed, type of diet, external deworming frequency, contact with stray dogs, and access to public parks. The interviews were conducted in person by trained staff, and the responses were cross-checked with clinical information and records available at veterinary clinics. The questionnaires were reviewed promptly after administration to maintain data quality, and those with incomplete or inconsistent responses were excluded.

### Statistical analysis

Statistical analysis was conducted using IBM SPSS Statistics v.26.0 (IBM Corp., NY, USA). Associations between qualitative variables and *E. canis* presence were evaluated using the chi-square test. Logistic regression was used to estimate odds ratios (ORs) and 95% confidence intervals (95% CIs) to identify potential risk factors. A factor was considered to represent a risk when p < 0.05 and both the OR and the lower limit of the 95% CI were greater than 1. The Benjamini–Hochberg correction was applied to control for type I error arising from multiple comparisons. Furthermore, the Hosmer–Lemeshow test was used to assess model fit. Hematological parameters were compared between seropositive and seronegative dogs using the non-parametric Mann–Whitney U test. Notably, the analysis did not consider the exact geographical origin of the animals, as many frequently moved between districts. Cases with incomplete or missing data were also excluded from the analysis.

## RESULTS

### Overall seroprevalence and epidemiological associations

The overall prevalence of *E. canis* among the dogs evaluated was 51.3% (95% CI: 46.7%–55.8%). Sex and breed showed no statistically significant association with seropositivity (p > 0.05) (Table 1). In contrast, dogs younger than 1 year of age had a 1.46 times higher risk of infection than those older than 1 year (p < 0.05; OR = 1.46; 95% CI >1). Similarly, animals without external deworming had a 1.99 times higher risk, whereas dogs fed homemade food or in frequent contact with stray dogs had 2.26- and 4.33-times higher risks, respectively (p < 0.05; OR > 1; 95% CI > 1). The presence of ticks and access to public parks were associated with a lower risk of seropositivity to *E. canis* (OR and 95% CI < 1; p < 0.05), suggesting that these factors may have a protective effect against infection.

**Table 1:** Factors associated with *Ehrlichia canis* in dogs from the city of Trujillo (n = 462), based on epidemiological factors.

Study variable	Positive		Negative		OR	CI (95%)	p-value*

	n	%	n	%			
Sex					1.11	0.77–1.60	0.56
Male	118	52.7	106	47.3			
Female	119	50	119	50			
Age					1.46	1.01–2.12	< 0.05**
< 1 year	116	56.6	89	43.4			
> 1 year	121	47.1	136	52.9			
Breed					1.26	0.87–1.83	0.21
Mixed	140	53.8	120	46.2			
Pure	97	48.1	105	51.9			
Presence of ticks					0.13	0.08–0.24	< 0.01
Yes	151	41.9	209	58.1			
No	86	84.3	16	15.7			
External deworming					1.99	1.36–2.91	< 0.01**
No	111	61.7	69	38.3			
Yes	126	44.7	156	55.3			
Contact with stray dogs					4.33	2.92–6.42	< 0.01**
Yes	145	70.7	60	29.3			
No	92	35.8	165	64.2			
Type of food					2.26	1.17–4.39	< 0.05**
Homemade	31	68.9	14	31.1			
Commercial	206	49.4	211	50.6			
Access to the public parks					0.26	0.15–0.44	< 0.05
Yes	170	45.5	204	54.5			
No	67	76.1	21	23.9			

** Identified as a risk factor, OR= Odds ratio, 95% CI = Confidence interval at 95%,

* chi-square non-parametric test

### Clinical signs associated with infection

Regarding the clinical findings (Table 2), dogs with anemia had a 4.12-fold higher risk of *E. canis* infection than those without this sign. Similarly, lethargy and anorexia were associated with 5.55- and 4.24-fold increased risks, respectively. Fever, lymphadenopathy, and epistaxis were significantly associated with infection (p < 0.05; OR > 1; 95% CI > 1), with 5.52-, 3.46-, and 2.50-fold higher risks, respectively. In contrast, other signs, such as lameness, uveitis, hypersalivation, and ocular discharge, were not significantly associated with the disease (p > 0.05; OR < 1) and were infrequent in the studied sample.

**Table 2:** Clinical signs associated with *Ehrlichia canis* in dogs from the city of Trujillo (n = 462)

Study variable	Positive		Negative		OR	CI (95%)	p-value*

	n	%	n	%			
Anemia					4.12	2.79–6.07	< 0.01**
Yes	153	68.9	69	31.1			
No	84	35.0	156	65.0			
Anorexia					4.24	2.39–7.53	< 0.01**
Yes	61	78.2	17	21.8			
No	176	45.8	208	54.2			
Lethargy					5.55	3.67–8.37	< 0.01**
Yes	188	67.1	92	32.9			
No	49	26.9	133	73.1			
Fever					5.52	3.71–8.23	< 0.01**
Yes	165	71.4	66	28.6			
No	72	31.2	159	68.8			
Lymphadenopathy					3.46	2.36–5.07	< 0.01**
Yes	149	66.8	74	33.2			
No	88	36.8	151	63.2			
Epistaxis					2.50	1.21–5.17	< 0.05**
Yes	27	71.1	11	28.9			
No	210	49.5	214	50.5			
Lameness					1.31	0.59–2.93	0.50
Yes	15	57.7	11	42.3			
No	222	50.9	214	49.1			
Uveitis					1.59	0.38–6.75	0.52
Yes	5	62.5	3	37.5			
No	232	51.1	222	48.9			
Sialorrhea					0.51	0.17–1.57	0.24
Yes	5	35.7	9	64.3			
No	232	51.8	216	48.2			
Ocular discharge					0.95	0.46–1.94	0.88
Yes	16	50.0	16	50.0			
No	221	51.4	209	48.6			

** Identified as a risk factor, OR= Odds ratio, 95% CI = Confidence interval at 95%,

* chi-square non-parametric test

### Hematological alterations in seropositive dogs

**Figure 2:**
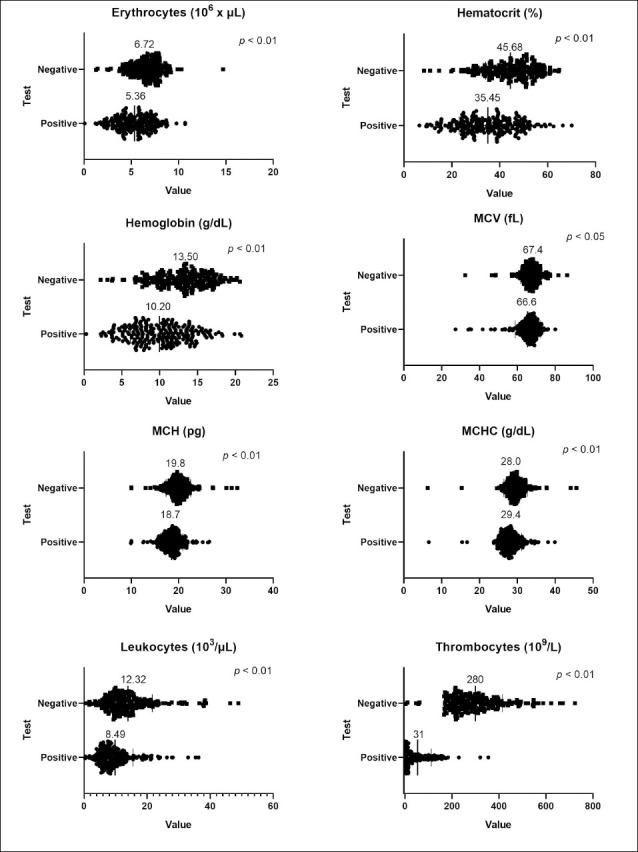
Mann–Whitney U test results (p < 0.05) and distribution of blood parameters (erythrocyte count, hematocrit, hemoglobin, mean corpuscular volume mean corpuscular hemoglobin, mean corpuscular hemoglobin concentration, and thrombocytes) in dogs that tested positive and negative for *Ehrlichia canis*. The mean of each group for each analyzed parameter is displayed.

Hematological analysis revealed significant differences between positive and negative dogs, as evaluated using the Mann–Whitney U test. The mean erythrocyte count was significantly higher in seronegative animals (6.72 × 10^6^/μL) compared to seropositive ones (5.36 × 10^6^/μL) (p < 0.01). Similarly, hematocrit levels averaged 45.68% in negative dogs and 35.45% in positive dogs (p < 0.01). Other hematological parameters, such as hemoglobin, MCV, MCH, leukocyte count, and platelet count, were significantly different. Figure 2 shows the average values for these parameters.

## DISCUSSION

### Vector importance and public health relevance

Canine ehrlichiosis is a disease transmitted by ticks of the genus, *specifically R. sanguineus*, which are of considerable public health importance because they can transmit other zoonotic diseases to humans [15, 16]. A study in Chiclayo, with climatic conditions similar to Trujillo, molecularly identified this ectoparasite in dogs, revealing genetic similarity to *R. sanguineus* from Brazil and Colombia [17]. However, this condition manifests with nonspecific clinical signs and hematological findings in dogs, with thrombocytopenia among the most common laboratory abnormalities [18]. For this reason, clinical diagnosis remains a challenge, particularly in the Peruvian coastal region, which experiences two distinct seasons (summer and autumn), influencing the epidemiological behavior of the disease.

### Potential for selection bias

Due to the sampling strategy employed, there is a potential for selection bias resulting from the preferential inclusion of dogs presenting compatible clinical signs or tick infestation. Although this approach increased the likelihood of detecting positive cases, it may also have led to an overestimation of the actual seroprevalence of *E. canis* in the general canine population. Therefore, the results should be interpreted with caution and considered representative mainly of animals with clinical suspicion of ehrlichiosis.

### Comparison with global and national prevalence

More than half of the analyzed dogs tested positive for *E. canis* antibodies in their blood samples, indicating a higher prevalence than in studies conducted in other parts of the world. For instance, studies in Bangladesh, Argentina, and Myanmar reported prevalence rates of 6.9%, 6.7%, and 0.75%, respectively [19–21]. The lower prevalence reported in Argentina may be explained by lower temperatures in Buenos Aires than in Trujillo, as well as by differences in owner practices and policies regarding pet health. Similarly, in Myanmar, molecular tests also yielded a low prevalence, suggesting that more specific methods, capable of detecting active infections, tend to reflect lower true prevalence rates. A similar pattern is observed in Bangladesh, where the reported low prevalence may also be related to the type of test used, despite favorable temperatures and humidity for the proliferation of the *E. canis* vector.

In Perú’s capital, Lima, rates ranging from 5.14% to 74% have been reported [22, 23]. Another study conducted in the city of Huánuco reported a prevalence very similar to that observed in the present study (51.3%) using the same diagnostic method [24]. In contrast, a study in the city of Iquitos reported a much lower prevalence (19.6%) using molecular methods [25].

### Environmental and epidemiological influences

These differences may be attributed to epidemiological factors related to the distribution of ticks, which thrive in warm environments such as those in Trujillo, especially between December and March, as well as to their ability to survive through hibernation under unfavorable conditions. According to a previous study [26], ticks can reduce their metabolic activity below 10°C or survive without feeding for up to 40 weeks. In this context, the northern coast of Perú does not experience temperatures below 10°C during the winter, allowing these ticks to remain active year-round and maintain a continuous life cycle, especially in stray dogs and in those who interact with them. Although there are no studies on the density of stray dogs in the city of Trujillo, a large number of these animals are commonly observed in parks or roaming the streets; moreover, many are frequently abandoned by their owners. Drug resistance or bacterial sensitivity and the frequency of deworming in dogs may also be key factors influencing the spread of the disease [27, 28]. Considering the above, it is common for pet owners in Perú to administer medications to their animals due to the lack of regulations restricting the sale of drugs to non-veterinarians. Such practices may contribute to the development of resistance in ectoparasites, hindering efforts to eradicate or control *E. canis*.

### Diagnostic complexity and differential considerations

The diagnostic challenge of *E. canis* in clinical settings is further complicated by its similarity to primary immune-mediated thrombocytopenia (pITP), especially when hematological results show thrombocytopenia. In this study, dogs aged 1 year were identified as being at higher risk, consistent with previous findings that younger animals have a higher probability of infection [29]. Clinical signs, such as anorexia, weight loss, lymphadenitis, and ocular discharge, were frequently observed, although the latter was not as prominent in this sample. Conversely, dogs with pITP also tend to present with epistaxis, often to a greater degree than those infected with *E. canis* [30, 31].

### Tick presence, environmental exposure, and confounding

Although there was a strong association between tick presence and infection, it was not statistically identified as a risk factor. This finding aligns with that of a previous study conducted in Lima (Perú) [23] and could be explained by the fact that some dogs may have been dewormed but still developed the disease. The observed association may also be due to higher tick activity during warmer periods and the tendency for *E. canis* prevalence to rise in regions with low annual rainfall, such as Trujillo [32]. Future studies could provide more insight into the behavior of this disease across both seasons on the Peruvian coast.

### Role of stray dogs, deworming practices, and socioeconomic factors

Contact with stray dogs, as shown in other studies, was identified as a risk factor for ehrlichiosis transmission because these animals may serve as reservoirs of infection [24]. Similarly, the absence of external deworming confirmed its relationship with infection, as reported in a previous study [19]. Although access to public parks was included as a variable, it was not associated with the disease and did not represent a direct risk factor for the disease. This suggests that the presence of *E. canis*-carrying ticks in parks does not significantly affect dewormed dogs. An interesting finding of this study was that dogs fed exclusively homemade food had a higher risk of infection. This may be linked to the socioeconomic status of families, as homemade food is more common in low-income households, where pets often receive less attention to nutrition and health care [33].

Although tick presence and access to public parks were significantly associated with seropositivity, both factors had ORs of 1. This seemingly paradoxical result could reflect a spurious protective effect, possibly influenced by confounding variables, such as a higher frequency of ectoparasite control in dogs that visit public spaces or a higher level of health awareness among their owners. These findings highlight the need for further analytical studies to account for these variables and confirm their true nature.

### Clinical and hematological correlates

Consistent with this study’s findings, dogs with anemia, fever, thrombocytopenia, leukopenia, and epistaxis are more likely to be infected with *E. canis*. These conditions help explain the significant differences in blood parameters observed between positive and negative dogs. However, age and sex did not appear to influence or be associated with the disease [22, 34, 35]. Nonetheless, other research reports conflicting results, suggesting that one sex (male or female) may be more prone to infestation [36, 37]. This variability may largely depend on sample representation and differences in care and sanitary management by owners of either sex.

Breed was not associated with disease presence, although mixed-breed dogs might be more predisposed, as suggested by previous studies [22, 38]. This might be because mixed-breed dogs typically receive less healthcare and have more frequent contact with stray animals. Similarly, corneal opacity was not a common sign in dogs testing positive for *E. canis*, contrary to findings in other studies, which also report hindlimb edema as a common symptom [37]. Although ocular lesions can be early indicators of ehrlichiosis [39], the diagnostic value of ocular lesions and the significance of ocular discharge remain uncertain.

### Joint and musculoskeletal manifestations

Lameness related to polyarthritis was also not commonly observed in this study’s sample, although it can occur during early infection [40]. Experimental studies have shown that *E. canis*-infected dogs rarely exhibit mononuclear cells, neutrophils, *E. canis* morulae, or macrophage reactivity in synovial fluid [41]. However, *E. canis* morulae were identified in only a few cases in which lameness was observed [42]. These findings suggest that the pathophysiology of this disease and its impact on joint tissues remain to be understood.

### Hematological alterations and immune response

Other studies have also reported significant differences in hematological parameters, with conditions such as neutropenia, pancytopenia, leukopenia, and thrombocytopenia being commonly observed, especially the latter, which is a strong positive predictor of infection [43]. However, in some regions, the prevalence of *E. canis* may exceed 80% even in dogs without evident clinical or hematological abnormalities [44]. This suggests that a dog’s health status and immune response may greatly influence disease severity, with multiple factors affecting its aggressiveness.

### Influence of treatment status

Notably, none of the dogs in this study had received treatment prior to blood sampling. Doxycycline is the antibiotic of choice for the treatment of ehrlichiosis in Perú. This drug causes changes in the hematological profile of infected dogs, particularly reductions in leukocytes and lymphocytes and increases in monocytes, eosinophils, and platelets [45]. B lymphocytes may temporarily increase, followed by a later decrease [46]. If this variable is not properly controlled, these fluctuations could significantly alter final hematological profiles and introduce bias in data analysis.

### Differential diagnosis with other vector-borne diseases

A study conducted in Panama reported that dogs infected with *E. canis* commonly present with thrombocytopenia, anemia, macroplatelets, and leukopenia, whereas those infected with *Anaplasma platys* exhibit some of these alterations but in different proportions [47]. Similarly, another investigation found that *Babesia* spp. infections are more strongly associated with reductions in hematocrit and hemoglobin, as well as increased monocyte counts, compared with *E. canis*, which is also characterized by severe thrombocytopenia [48]. Therefore, evaluating parameters such as anemia, thrombocytopenia, and leukocyte differentials can be highly useful for veterinarians in distinguishing ehrlichiosis from babesiosis or anaplasmosis in clinical practice. Nevertheless, this should be complemented with reliable diagnostic testing, particularly in cases of coinfection with *Babesia* spp. and *Anaplasma* spp., where marked thrombocytopenia is also observed [43].

### Strengths and limitations of diagnostic methods

Rapid immunochromatographic tests are widely used in Latin America due to their accessibility and ease of use, though their cost remains a limitation for some pet owners. This study used the CaniV-4® test, which has a sensitivity of 97.6% and specificity of 99% for detecting *E. canis* [49], making it a reliable diagnostic tool in clinical settings with limited resources. However, this test only detects circulating antibodies, which can persist for months or even years after clinical resolution or treatment, and may not reflect an active infection [1, 50]. It is true that cross-reactivity among *Ehrlichia* species is possible [51, 52]. Consequently, the estimated seroprevalence in this study should be interpreted as an indicator of prior immune exposure rather than evidence of current infection, a significant diagnostic limitation when detection is based solely on serological methods.

Techniques such as polymerase chain reaction (PCR) and indirect immunofluorescence, the latter considered the “gold standard”, offer nearly 100% sensitivity and specificity [53]. However, these diagnostic resources are not widely available in many areas, such as Trujillo, where most veterinary clinics lack such capabilities. Additionally, the variability in clinical signs is influenced by external and physiological factors, which must be carefully assessed by professionals [54]. Integrating these clinical findings with complementary tests enables a definitive diagnosis and supports a specific and comprehensive treatment of the disease.

### One health implications and epidemiological importance

The high prevalence of *E. canis* in dogs in the city of Trujillo highlights the need to address vector-borne diseases within a comprehensive One Health perspective. The findings reflect not only a veterinary challenge but also an indicator of the shared environmental and health risks among humans, animals, and the ecosystem [55, 56], particularly involving veterinarians, pet owners, stray dogs, and public parks. Moreover, the persistence of *E. canis* suggests favorable conditions for vector proliferation, possibly associated with climatic factors, urban distribution, and close interactions between domestic dogs and humans [57, 58]. Therefore, the control of this disease should be framed within a multidisciplinary approach that includes integrated epidemiological surveillance, vector management, and community health education.

### Public health relevance and need for coordinated action

In this context, ehrlichiosis carries direct public health implications, as some *Ehrlichia* species are zoonotic, and the vector can also transmit other medically relevant pathogens [2, 59, 60]. Implementing coordinated programs involving veterinarians, human healthcare professionals, and environmental authorities would allow the identification of risk areas, optimize entomological surveillance, and reduce human and animal exposure to infected ticks. Strengthening multidisciplinary cooperation and maintaining continuous monitoring of ehrlichiosis in canine populations can serve as an early indicator of health status in urban environments such as Trujillo.

## CONCLUSION

This large-scale cross-sectional study demonstrated a high seroprevalence of *E. canis* (51.3%) among domestic dogs in Trujillo, Perú, confirming widespread exposure to the pathogen in a region characterized by climatic and environmental conditions favorable to the year-round persistence of *R. sanguineus*. Several strong epidemiological and clinical predictors of infection were identified, including younger age (<1 year), lack of external deworming, feeding of homemade diets, and frequent contact with stray dogs. Clinically, anemia, lethargy, anorexia, fever, lymphadenopathy, and epistaxis were significantly associated with seropositivity, while hematological analysis revealed marked reductions in erythrocytes, hemoglobin, hematocrit, leukocytes, and platelets in infected dogs. Collectively, these findings reinforce the diagnostic value of combined clinical–epidemiological assessment in settings where molecular tests remain limited.

From a practical standpoint, the results highlight urgent needs in routine veterinary practice: consistent external deworming, owner education on tick-control, improved nutritional oversight, and heightened clinical suspicion for ehrlichiosis in dogs presenting with compatible signs. The study also underscores the importance of community-level management of stray dog populations, as these animals are likely reservoirs that maintain pathogen circulation. Furthermore, recognizing anemia and thrombocytopenia as strong laboratory markers can help guide clinical decision-making where advanced diagnostics are unavailable.

A key strength of this study lies in its large sample size, standardized clinical assessment, and integration of epidemiological, serological, and hematological data across multiple districts. However, limitations should be acknowledged. The use of serological testing may overestimate past exposure rather than active infection, and sampling dogs with suspected ehrlichiosis may introduce selection bias. The absence of molecular confirmation restricts conclusions about circulating strains, and the lack of detailed data on stray dog density, acaricide resistance, and household socioeconomic factors limits comprehensive interpretation of transmission dynamics.

Future research should incorporate molecular diagnostics such as PCR to distinguish active from past infections, characterize circulating genotypes, and identify possible coinfections with *Babesia* spp. or *Anaplasma* spp. Longitudinal studies evaluating seasonal patterns, ectoparasite control practices, acaricide resistance, and human exposure risks would substantially enhance understanding of local transmission. One Health–oriented investigations involving veterinarians, pet owners, public health authorities, and environmental agencies are recommended to design integrated surveillance and control programs.

In conclusion, the high burden of *E. canis* in Trujillo reflects persistent vector presence, gaps in preventive care, and close interactions between domestic and stray dogs. Strengthening tick-control strategies, improving owner awareness, and expanding diagnostic capacity are essential steps toward reducing disease impact. Coordinated multisectoral actions rooted in the One Health framework will be crucial for mitigating the veterinary and public health implications of canine ehrlichiosis in northern Perú.

## DATA AVAILABILITY

All the generated data are included in the manuscript.

## AUTHORS’ CONTRIBUTIONS

RRR and LKQ: Conceptualization and design of the study. RM and JRP: Data collection. JRP: Data analysis and interpretation. RRR, LKQ, and RM: Laboratory work and data acquisitions. All authors have read and approved the final version of the manuscript.
